# Genetics of RA susceptibility, what comes next?

**DOI:** 10.1136/rmdopen-2014-000028

**Published:** 2015-04-15

**Authors:** James Ding, Stephen Eyre, Jane Worthington

**Affiliations:** Arthritis Research UK Centre for Genetics and Genomics, Centre for Musculoskeletal Research, Institute of Inflammation and Repair, Manchester Academic Health Science Centre, The University of Manchester, Manchester, UK

**Keywords:** Autoimmune Diseases, Rheumatoid Arthritis, Inflammation, Arthritis

## Abstract

Genome-wide association studies (GWASs) have been used to great effect to identify genetic susceptibility loci for complex disease. A series of GWAS and meta-analyses have informed the discovery of over 100 loci for rheumatoid arthritis (RA). In common with findings in other autoimmune diseases the lead signals for the majority of these loci do not map to known gene sequences. In order to realise the benefit of investment in GWAS studies it is vital we determine how disease associated alleles function to influence disease processes. This is leading to rapid development in our knowledge as to the function of non-coding regions of the genome. Here we consider possible functional mechanisms for intergenic RA-associated variants which lie within lncRNA sequences.

Rheumatoid arthritis (RA) is a polygenic autoimmune disease that is characterised by chronic inflammation of the synovial joints, and associated with the presence of anticitrullinated protein antibodies (ACPA). For over 40 years it has been well established that there is a strong association between susceptibility to RA and human leucocyte antigen (HLA) DRB1 alleles that share a consensus sequence, known as the ‘shared epitope’.^1^ Spanning position 70–74 of the β subunit of the HLA DR molecule, this association accounts for approximately 40% of the genetic component of susceptibility of RA, and has recently been refined following imputation of amino acid sequences from high-density single nucleotide polymorphism (SNP) data.^2^ It is only since the introduction of SNP-based genome-wide association studies (GWAS) in 2007 that an extensive list of additional genetic risk loci, have been associated with RA.^3^ Currently, over 100 risk loci have been defined, explaining approximately half of the genetic variance in RA.^4^ ^5^

The challenge in this post-GWAS era is to interpret and translate these genetic findings, defining how they influence disease susceptibility and outcome. Signals from GWAS typically comprise large numbers of highly correlated SNPs defining regions that contain, on average, four genes, thus the remaining challenge is to identify for each locus the functional variant(s), the genes on which they act and the specific cell type in which dysregulation contributes to disease susceptibility.

In addition to providing insights into RA pathogenesis, understanding genetic susceptibility at this level has the potential to impact on both drug discovery and the optimal implementation of established therapies through a greater understanding of disease heterogeneity. Thus far, functional studies to further characterise implicated genetic regions in disease have focused almost exclusively on associated variants in protein-coding genes, however, these account for only a small minority of associated loci. Evidence suggests that for RA and many other complex diseases the majority of associated variants map outside protein coding regions.

## Intergenic RA loci

In contrast to monogenic traits, an enrichment analysis performed using data from over 100 GWAS publications has shown that SNPs associated with complex traits are intergenic and do not map to protein sequences.^6^

Traditionally, the functional implications of intergenic RA loci have been attributed to the nearest protein-coding gene known to have immunological relevance. This assumption, that genetic polymorphisms alter the expression of neighbouring genes by affecting cis-regulatory elements, such as enhancers and promoters, has been demonstrated to be misguided at a number of loci. For example, an association between the chromosome 16p13 region and a number of autoimmune diseases has been shown. This association was originally attributed to *CLEC16A*, with the majority of disease associated SNPs found within the transcribed region of this gene, especially within intron 19. *CLEC16A* is expressed in a variety of immune relevant cells and contains an immunoreceptor tyrosine-based activation motif. Functional studies have, however, revealed a long range chromatin interaction between intron 19 of *CLEC16A* and regulatory sequences that affect the expression of *DEXI*, found ∼140 kb from the SNPs.^7^ The focus of research at this locus has now moved to *DEXI* as an autoimmune candidate gene. A similar example, where the SNPs in question are intergenic, rather than intronic, can be found at the chromosome 1p13 region, which is associated with low-density lipoprotein cholesterol and myocardial infarction. Here a disease associated SNP disrupts a transcription factor binding site found between *CELSR2* and *PSRC1*, which inhibits the expression of *SORT1*, found ∼35 kb from the putative causal SNP.^8^

Evidence is now emerging that the variants most strongly associated with autoimmune disease (lead SNPs) are enriched in areas of the chromosome that; are open and active, are defined by epigenetic marks, regulate gene transcription, and act in a cell-specific manner. Indeed, a recent study of 21 autoimmune diseases by Farh *et al*,^9^ described an enrichment of putative causal SNPs in immune-cell enhancers. The majority (∼60%) of these SNPs, associated with RA and 20 other autoimmune diseases, map to enhancer elements that are active in lymphoblasts, lymphocytes and macrophages. The RA-associated enhancers are enriched for; cell specificity (T and B cells), activity in response to stimulation, and size (large ‘super-enhancers’). Furthermore, they are associated with the expression of non-coding RNAs (enhancer-derived RNAs, eRNAs also known as elncRNAs). This was shown to be true within the subset of RA-associated super-enhancers in the analysis by Farh *et al*, but is best illustrated by Hnisz *et al*^10^ in one of the first papers to describe super-enhancers. Super-enhancers, consist of clusters of enhancer elements that play a key role in defining cell identity and are associated with a 24.3-fold enrichment of RNA, compared to typical enhancers. Fahr *et al* found a 3.2-fold enrichment of RA SNPs in super-enhancers compared to typical enhancers and speculated that these SNPs could elicit subtle and stimulation specific changes in gene expression. Defining the function of these ncRNA expressing regions will be pivotal if we are to translate GWAS findings into mechanisms of susceptibility.

By recruiting various proteins, enhancers mediate chromosomal interactions, enabling the assembly of transcriptional machinery at promoter regions. It is well established that these regulatory elements do not necessarily affect the transcription of neighbouring genes, can function over large distances and generally affect the expression of more than one gene.^11^ One of the hallmarks of these regulatory regions is accessible chromatin, as indicated by regions of DNase I hypersensitivity. Other markers that offer greater specificity include chromatin modifications: Enhancer regions are associated with high levels of histone 3 lysine 4 monomethylation and dimethylation, relative to trimethylation (H3K4me1, H3K4me2 and H3K4me3, respectively), with active enhancers characterised by histone 3 lysine 27 acetylation (H3K27ac).^12^

Lead disease-associated variants, situated as they are in gene regulatory regions, may have a number of functional consequences that contribute to increased risk of RA. These include disruption of transcription factor binding sites and chromatin interactions, as at the *SORT1* and *DEXI* loci, as well as other mechanisms of disrupting regulatory features such as enhancers, silencers, promoters and insulators. Examples include potential impacts on the deposition of epigenetic markers and RNA binding. It is also possible for non-coding SNPs to affect the function of non-coding RNA transcripts, such as those of eRNA of lncRNA, by altering their sequence or expression pattern.

## lncRNA

lncRNA are traditionally defined by an arbitrary lower limit of 200 bases, with over 100 000 transcripts identified in humans.^13^ Their diverse functions and characteristics have only recently attracted the attention of the scientific community (see box 1 for a description of various lncRNA categories). Further classification of lncRNA has focused on genomic content, with over a third of transcripts found in intergenic regions (long intervening non-coding RNA, lincRNA). While advances in RNA sequencing (RNA Seq) techniques can account for many of the lncRNA species identified to date, a large number of lincRNA have been identified by having a similar chromatin signature to that of other actively transcribed genes. Typically, trimethylation of histone 3 lysine 4 is (H3K4me3) is observed at the promoter, with histone 3 lysine 36 trimethylation (H3K36me3) found along the transcribed length.

Box 1 **Summary of lncRNA features and classification.**

Long non-coding RNA (lncRNA)

- Defined as over 200nt in length. Generally transcribed by RNA Polymerase II, spliced and polyadenylated, as for protein-coding genes.
- lncRNA are often characterised according to genomic position for example, exonic, intergenic (lincRNA), etc.
- Other, functional categories have been proposed.
  - For example, activator RNAs stimulate transcription
- Enhancer-derived RNAs (eRNAs) fall into two categories
  - 1D-eRNAs: Derived from enhancers that are transcribed unidirectionally.
  - 2D-eRNAs: Derived from enhancers that are transcribed bidirectionally.
  - More common and not polyadenylated.

lincRNAs are typically transcribed by RNA Polymerase II and often undergo polyadenylation and splicing, just like protein-coding transcripts.^14^ This is, however, not the case for some eRNAs, which are subdivided according to whether they are unidirectionally or bidirectionally transcribed (1D-eRNA, 2D-eRNA). eRNA are generally unspliced, with only the less common 1D-eRNAs undergoing polyadenylation.

In order to identify RA-associated loci where increased risk is likely to be mediated by lncRNA we have compiled a list of RA-associated regions that contain lincRNA and do not contain protein-coding genes. Regions of high linkage disequilibrium (encompassing all SNPs in linkage disequilibrium, r^2^=0.8 with the lead GWAS variant) were defined around the 102 regions associated with RA and computationally intersected with a database of lincRNA (using bedtools).^15^ This was performed with all lncRNAs annotated as intergenic in the lncRBase database.^13^ Of the 25 regions returned, 12 contained lncRNA that originate from Human Body Map RNA sequencing data,^16^ with the provenance of the remaining lncRNAs generally being Expressed Sequence Tags (ESTs) found in the NONCODE database.^17^ Manual curation, using UCSC genome browser,^18^ resulted in a list of 9 lncRNA containing RA regions that are sufficiently distant from protein-coding transcripts as to make any direct influence on their expression an unlikely consequence of disease-associated variants. A list of these regions is provided in table 1.

**Table 1:** Intergenic RA-associated regions that overlap lincRNA

RA-associated region	Lead SNP	Gene	Expl.H^2^	OR	Overlapping lincRNA
chr1:38614865–38644861	rs12140275	POU3F1	0.039	1.11	lnc-UTP11L-2
chr2:100806512–100835734	rs9653442	AFF3	0.064	1.11	lnc-NMS-1
chr4:26085478–26128710	rs11933540	SMIM20	0.101	1.15	lnc-RBPJ-1
chr6:137959233–138006504	rs17264332	TNFAIP3	0.100	1.17	lnc-OLIG3-1 and lnc-TNFAIP3-3
chr6:159489789–159515309	rs2451258	TAGAP	0.041	1.10	lnc-FNDC1-7
chr8:102451261–102469182	rs678347	GRHL2	0.034	1.08	lnc-ZNF706-5
chr8:129540462–129571140	rs1516971	TMEM75	0.044	1.15	lncTMEM75-2
chr15:69984460–70010647	rs8026898	RPLP1	0.079	1.15	lnc-RPLP1-2
chr16:86005836–86021627	rs13330176	IRF8	0.064	1.12	lnc-IRF8-5

Included is the associated region (r^2^=0.8), lead SNP, closest protein-coding gene (Gene), explained heritability (Expl.H^2^, %) OR and overlapping lincRNA (LNCipedia nomenclature).^19^ Explained heritability and ORs are taken from Okada *et al.*^5^

RA, rheumatoid arthritis; SNP, single nucleotide polymorphism.

Fully characterising these lncRNA and the impact RA-associated variants have on their function will be an important area of research aimed at elucidating mechanisms of disease susceptibility. Generalised functional models for lncRNA are difficult to impose due to the variety of mechanisms observed, however, most functional lncRNA seem to act as scaffolds, regulating the formation of complexes that can be composed of protein, DNA and other RNA.^14^ ^20^ In brief, some of the complexes that lncRNA have been implicated in include the mediator complex^21^ and histone modifiers, such as Polycomb Repressive Complex 2 (PRC2).^22^ Frequently, lncRNA are implicated in regulating the transcription of other genes, in *cis* and in *trans.*^23^ Further complications include evidence that the act of transcription from certain lncRNA loci can be functional regardless of the transcript produced.^24^

Evidence supporting a role of lincRNAs in autoimmune disease is beginning to accumulate as this field attracts the interest of the scientific community. This evidence includes observations of differential regulation of lincRNA in pathways relevant to RA. For example in CD14+ monocytes isolated from patients with RA and stimulated with tumour-necrosis factor-α or interleukin 6, two non-overlapping subsets of lincRNA were found to be differentially regulated (60 and 25 lincRNAs, respectively).^25^ A separate study has linked changes in lincRNA expression to genotype using GWAS data, introducing the concept of lincRNA expression quantitative trait loci (lincRNA eQTLs). In general, these eQTLs were tissue specific and affected only lincRNAs and not a downstream regulated protein coding gene.^26^ As for enhancers, these findings encourage a stimulus and tissue-specific approach to investigating the role of lncRNA in RA.

## Investigating lncRNA

Many online resources are available that enable the prioritisation of candidate lncRNAs based on publicly available data (table 2). These include tools to overlay disease-associated SNPs with lncRNA, investigate the provenance and expression of these lncRNAs and to examine other genomic features of these loci, such as methylation, chromatin modifications and transcription factor binding.

**Table 2:** Online lncRNA resources

Database	Nomenclature	size	Description of key features	Provenance of lncRNAs	Reference
lncRBase	hsaLB_AN_74656	133 361	Comprehensive database categorising lncRNA according to genomic context. Integrates, coding potential, expression data, associated genomic elements and publications	lncRNAdb, Broad Institute, Ensembl, NONCODE, H-InvDB	13
lncipedia	lnc-SMUG1-7	113 513	Grouping of isoforms that share at least one exon. Integrates prediction of secondary structure, locus conservation and coding potential	lncRNAdb, Ensembl, Gencode, RefSeq, NONCODE, Broad Institute and two further RNA Seq publications	19
NONCODE	n343067/NONHSAT028510	95 135	ncRNA database with detailed information regarding provenance and expression	RefSeq, literature mining and specialised databases	17
lncRNASNP	lnc-SMUG1-7	32 108	Lists overlapping miRNA binding sites and SNPs, predicting the influence of SNPs on secondary structure and miRNA binding and providing corresponding miRNA expression data	lncipedia, dbSNP and stringency filtering	27
Ensembl	ENSG00000228630	23 498	Integrative database of genome annotation with detailed information regarding provenance	Computational prediction from ESTs and chromatin marks	28
Gencode	ENSG00000228630	15 877	Integrative database including information on DNA methylation, occupancy and chromatin state, along with RNA expression and binding	HAVANA manual annotation and Ensembl annotation pipeline, mostly validated	29
Havana	OTTHUMT 00000328662	14 396	Manual genomic annotation supported by transcriptional evidence	Manual annotation	29
Broad Institute	TCONS_00079054	14 353	Expression levels in 24 tissues and cell types, stringent set of 4662 lincRNA	RNA Seq in 24 tissues and cell types	16
ChIPBase	lncRNA2314-1	10 559	Integrates chromatin immunoprecipitation sequencing data with lncRNA occupancy	Ensembl, refseq, UCSC, lncRNAdb and various publications	30
RefSeq	NR_047517.1	6917	Non-redundant database of annotated sequences	Data submitted to the International Nucleotide Sequencing Database	31
lincPoly	XLOC_000000*	4662	Similar to lncRNASNP, with SNPs categorised according to phenotype and conservation data integrated in the place of miRNAs	Stringent set from Broad Institute	32
lncRNAdb	HOTAIR	166	Manually curated database ofexperimentally validated, functional lncRNAs	Literature mining	33

This selection of resources is not exhaustive, but presents a broad spectrum of available and up to date tools. Included is an example of the nomenclature preferred by each database, with all entries referring to the lncRNA HOTAIR.

*HOTAIR is not found in lincPoly.

Once genetically-associated lncRNA are identified and investigated bioinformatically, a wide variety of molecular biology techniques can be used to interrogate their function. In many cases the techniques are similar to those used to investigate the transcripts of coding genes, however, several additional considerations exist.^34^ It is important that experiments are designed in order to distinguish between loci that transcribe functional RNA, and loci where the process of transcription is important for the function of underlying regulatory elements. Perturbation experiments, including depletion and overexpression, are valuable for assessing RNA function, with genetic engineering a necessary component of experiments designed to investigate the role of genomic elements.

Expression of lncRNAs can be investigated, as for any other transcript, by quantitative PCR (qPCR), microarray, RNA sequencing (RNA-Seq) or fluorescence in situ hybridisation (FISH). A modification of the protocol used to create probes for FISH can be used to simultaneously generate tools for depleting lncRNA expression.^35^ Commercial locked nucleic acids (LNAs) are also available for lncRNA depletion, with overexpression easily achieved using vector-based systems, similar to those used for protein-coding genes.

Several immunoprecipitation techniques exist that enable the identification of lncRNA-binding partners (examples of these techniques and references to their use are found in table 3). Given the propensity of lncRNAs to act as scaffolds, these experiments are of clear importance. When lncRNA are enhancer-derived, it is also wise to consider investigating their influence on DNA–DNA interactions. A variety of techniques exist for identifying and investigating enhancers and may well form the basis of further experiments.^36^

**Table 3:** Immunoprecipitation techniques for investigating lncRNA

Acronym	Technique	Discriminating features	Reference
CHART	Capture hybridisation analysis of RNA targets	Tiled oligonucleotides against lncRNA, yielding bound DNA and protein	37
ChIRP	Chromatin isolation by RNA purification	Pool of ∼20nt probes and gluteraldehyde crosslinking. Yields bound DNA and protein	38
CLASH	Crosslinking, ligation and sequencing of hybrids	Small pool of ∼20nt probes. Partial RNaseH treatment during elution. Yields bound RNA	39
iCLIP	Individual nucleotide resolution UV-crosslinking and immunoprecipitation	Antibody used against protein of interest, with interacting lncRNA nucleotide interrogated by sequencing	40
RAP	RNA antisense purification	Large pool of ∼120nt probes. Yields bound DNA and RNA	41 42
RIP	RNA immunoprecipitation	Antibody used against protein of interest, with presence of lncRNA interrogated. No cross-linking	43
ChRIP	Chromatin RNA immunoprecipitation	Antibody used against modified chromatin, with presence of lncRNA interrogated	44
ChOP	Chromatin oligo-affinity precipitation	Single ∼80nt probe, yielding interacting DNA	45

All methods use biotinylated oligonucleotides as probes against the lncRNA of interest and formaldehyde cross-linking, unless otherwise stated.

## Potential impact

**Figure 1:**
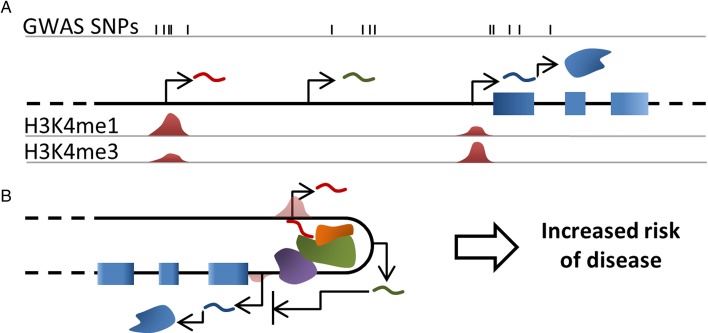
Graphical summary. (A) Genome-wide association studies single nucleotide polymorphism (GWAS SNPs) for complex diseases are mainly found outside of protein coding regions, and often overlay lncRNAs and/or enhancer elements. In this example, SNPs are found overlapping and proximal to three transcripts (curved lines); including two lncRNA, one of which is enhancer derived (red) and a mRNA (blue). (B) A detailed investigation of how these variants contribute to increased disease risk will have a profound impact on our understanding of the disease and is likely to implicate enhancers and lncRNA. lncRNAs (eg, green curved line) often function by regulating the transcription or translation of protein coding genes. One model of how this may occur is the mediation of chromatin interactions (eg, red curved line), which may involve the recruitment of chromatin modifiers, transcriptional activators or other proteins (green and orange forms, RNA pol II is represented by the purple form).

This success in identifying a large number of genetic associations with RA is yet to be translated into a thorough understanding of its aetiology and pathogenesis. The challenge we now face is to pinpoint the disease-associated variants and understand how they dysregulate normal biology and lead to the development of disease processes (figure 1). One of the emerging concepts in post-GWAS studies is the discovery of an enrichment of associated SNPs in intergenic regions, active in specific-cell types. We have described how bioinformatics analysis of RA loci implicates lncRNA and eRNA regions and propose that investigation of these loci may reveal novel insights into the pathogenesis of RA.

Thorough investigation of how disease-associated SNPs affect lncRNA, in a cellular and stimulation-specific manner, has the huge potential to link downstream genes, pathways and cellular outcomes empirically to disease causing variants. This is likely to implicate specific enhancer elements, regulatory targets and cell types in different diseases and will have an obvious impact on the understanding on complex disease aetiology, especially where these components are shared.
